# Stable, compounded bedaquiline suspensions to support practical implementation of pediatric dosing in the field

**DOI:** 10.5588/ijtld.22.0440

**Published:** 2023-03-01

**Authors:** R. Taneja, M. C. Nahata, J. Scarim, P. G. Pande, A. Scarim, G. Hoddinott, C. L. Fourie, R. K. Jew, H. S. Schaaf, A. J. Garcia-Prats, A. C. Hesseling

**Affiliations:** 1 Global Alliance for TB Drug Development (TB Alliance), New York, NY, USA; 2 Institute of Therapeutic Innovations and Outcomes, Colleges of Pharmacy and Medicine, The Ohio State University, Columbus, OH, USA; 3 JSAS Services Inc Tucson, AZ, USA; 4 Desmond Tutu TB Centre, Department of Paediatrics and Child Health, Faculty of Medicine and Health Sciences, Stellenbosch University, Tygerberg, South Africa; 5 Metro TB Complex, Department of Health, Pretoria, South Africa; 6 Institute for Safe Medication Practices (ISMP), Plymouth Meeting, PA, USA; 7 Department of Pediatrics University of Wisconsin School of Medicine and Public Health, Madison, WI, USA

**Keywords:** antitubercular, bedaquiline, liquid suspensions, drug storage stability, paediatric TB

## Abstract

**BACKGROUND:**

Bedaquiline (BDQ) tablets are indicated as part of a combination regimen for the treatment of multidrug-resistant TB in adults, adolescents and children. A dispersible tablet formulation is now approved but is not currently available in all settings. The aim of this study was to develop stable extemporaneous liquid formulations of BDQ that can be stored at room temperature or 30°C for several weeks, to support pragmatic pediatric dosing in the field and reduce wastage.

**METHODS:**

BDQ tablets were suspended in simple syrup and a sugar-free vehicle. Each 20 mg/mL formulation was stored at room temperature or 30°C for 30 days in amber dispensing bottles. Appearance, BDQ potency, pH and microbial counts were determined on Days 0, 15 and 30.

**RESULTS:**

The BDQ potency in both formulations remained at 98–101% of the theoretical concentration for 30 days. The appearance, pH and microbial count of sugar-free formulation did not change during the 30-day storage. The simple syrup formulation was stable for 15 days as microbial growth was observed on Day 30.

**CONCLUSIONS:**

BDQ may be prepared in syrup or sugar-free suspensions: syrup suspensions can be stored for 15 days at room temperature and 30C, whereas sugar-free suspensions can be stored for 30 days at room temperature and 30C. This information will support practical BDQ dosing for children in the field.

## Abstract (fr)

**CONTEXTE:**

Les comprimés de bédaquiline (BDQ) sont indiqués dans le cadre d’une association thérapeutique pour le traitement de la TB multirésistante chez l’adulte, l’adolescent et l’enfant. Une préparation sous forme de comprimé dispersible est désormais homologuée, mais elle n’est actuellement pas disponible dans tous les contextes. L’objectif de notre étude était de développer des préparations liquides extemporanées stables de BDQ, pouvant se conserver à température ambiante ou à 30°C pendant plusieurs semaines pour favoriser un dosage pédiatrique pragmatique sur le terrain et réduire les gaspillages.

**MÉTHODES:**

Des comprimés de BDQ ont été formulés en suspensions dans une préparation de sirop simple et dans une préparation sans sucre. Chaque préparation de 20 mg/ml a été conservée à température ambiante ou à 30°C pendant 30 jours dans des flacons ambrés. L’apparence, la puissance de la BDQ, le pH et le taux de bactéries ont été déterminés aux Jours 0, 15 et 30.

**RÉSULTATS:**

La puissance de la BDQ dans les deux préparations est restée à 98–101% de la concentration théorique pendant 30 jours. L’apparence, le pH et le taux de bactéries de la préparation sans sucre n’ont pas changé pendant la période de conservation de 30 jours. La préparation sous forme de sirop simple est restée stable pendant 15 jours, mais une croissance bactérienne a été observée au Jour 30.

**CONCLUSIONS:**

La BDQ peut être préparée en suspension dans une préparation de sirop ou une préparation sans sucre et être conservée pendant respectivement 15 et 30 jours à température ambiante ou à 30°C. Cette information favorisera un dosage pédiatrique pratique de la BDQ sur le terrain.

Multidrug-resistant (MDR-) and rifampicin-resistant TB (RR-TB) remain significant challenges in global health. As per the 2021 Global TB Report,^1^ 1.5 million people are affected by MDR/RR-TB, of which 22% were treated. In comparison, only 11% of the 115,000 MDR/RR-TB cases in children received treatment. When treated, outcomes in children with RR/MDR-TB are good, with favorable outcomes in at least 78%^2^ of the treated population. Despite this, relatively few children are treated for RR/MDR-TB each year, with only 3,398 starting treatment in 2018 and 5,586 starting treatment in 2019.^3^ Treatment with MDR-TB regimens are frequently hard to tolerate in children due to the complexity of regimens, duration of treatment, drug toxicity and the historical lack of child-friendly formulations.^2^

Bedaquiline (BDQ) has been approved by the US Food and Drug Administration (FDA) for the treatment of adult and pediatric patients aged ≥5 years, with ≥15 kg body weight, with confirmed or probable pulmonary MDR-TB. It is labelled for use with at least three other drugs to which patient’s isolate is susceptible in vitro, and four other drugs to which the isolate is likely to be susceptible when in vitro testing is unavailable.^4^ Studies have reported improved microbiological and clinical outcomes with the use of BDQ in adults, with MDR-TB or extensively drug-resistant TB (XDR-TB).^4^–^8^

Each BDQ tablet contains 120.89 mg of BDQ fumarate equivalent to 100 mg of BDQ.^4^ A20 mg BDQ tablet that can be dispersed in water was approved by the US FDA in May 2020 and is available through the Global Drug Facility.^9^ As per the dosing guidelines in the Field Guide of the Sentinel Project,^10^ the recommended dosage for children ranging from 3 kg to <30 kg body weight is 60 mg to 200 mg. Although a 20 mg scored tablet allows for flexibility when dosing small children, it has yet to be determined whether it corresponds with the best strength to optimally dose children across all ages.^11^

Four different methods of administration are outlined in the patient instruction for 20 mg tablets,^4^ which involve either dispersing the tablets in water or crushing the tablets, and then mixing the dispersion or crushed tablets with soft food or beverage. Once dispersed, the dispersion has to be consumed immediately and cannot be stored; therefore, each dose has to be prepared at the time of administration. This procedure must be repeated daily by the patient or caregiver, and the volume for 60–200 mg dose mentioned above would range from 5 mL to 10 mL to be added to food or beverage. This can be tedious for the patients and caregivers and leads to unnecessary wastage. The objective of this study was to develop two stable extemporaneous liquid formulations of BDQ 20 mg/mL, which could be compounded in a routine pharmacy or dispensary and provided to the caregivers. The concentration of 20 mg/mL allows for volumes of 3–10 mL for the dose range. These volumes can be measured with an oral syringe and administered directly without the need to mix with food or beverage. The vast majority of people with MDR-TB live in low- and middle-income countries (LMICs). Therefore, we conducted this study by utilizing easily accessible and affordable ingredients and minimal equipment to prepare the formulations to ensure maximal applicability to routine care in LMICs. Storage stability was investigated at room temperature (22–24°C) and at 30°C for at least 2 weeks.

## METHODS

### Preparation of bedaquiline formulation in simple syrup

Simple syrup was prepared by dissolving 255 g of cane sugar in 135 mL of hot distilled water by stirring. The syrup was allowed to cool to room temperature before use. Twenty BDQ 100 mg tablets were ground into a fine powder in a glass mortar and pestle. Fifteen mL of simple syrup were mixed with the powder to obtain a smooth mixture. An additional 81 mL of syrup was added in increasing amounts while mixing thoroughly. The total amount of simple syrup added to the mortar is 96 mL resulting in 100 mL of suspension. The contents were mixed well to obtain 100 mL of 20 mg/mL of a uniform BDQ suspension. This suspension was transferred into an amber 120-mL pharmacy dispensing bottle with a syringe adapter and child-resistant cap for storage. Step-wise preparation instructions are described in Table 1.

**Table 1:** Instructions for preparation of BDQ 20 mg/mL formulation in simple syrup

	Composition:	
Simple syrup vehicle		
Ingredient	Strength	Quantity
Sugar	65% w/w^*^	255 g
Distilled water	—	135 mL
BDQ sugar formulation, 20 mg/mL, 100 mL^†^		
Ingredient	Strength	Quantity
BDQ tablets	100 mg	20
Simple syrup	—	96 mL (qs 100 mL)

^*^ Instructions for preparing simple syrup (65% w/w) vehicle from cane sugar: 1 Weigh 255 g of food-grade cane sugar into a suitable container 2 Add 135 mL of hot distilled water and mix well until sugar is dissolved 3 Cool syrup to ambient room temperature

^†^ Instructions for 100 mL of 20 mg/mL BDQ formulation in simple syrup: 1 Grind 20 tablets of BDQ (100 mg each) to a fine powder in a glass mortar and pestle 2 Mix powder with a small amount (15 mL added using an oral syringe) of simple syrup vehicle to form a uniform paste 3 Add an additional 81 mL of syrup vehicle in increasing amounts while mixing thoroughly to bring total volume in the mortar to 100 mL 4 Transfer the final contents from the mortar into an appropriately sized amber bottle

w/w = weight by weight; BDQ = bedaquiline.

### Preparation of bedaquiline sugar-free formulation

Thick & Easy® Instant Food and Beverage Thickening Powder (Hormel Foods Sales LLC, Austin, MN, USA) is a widely available modified food starch that is used as a thickening agent in foods and liquids for patients with difficulty swallowing. Thick & Easy sugar-free vehicle was prepared by stirring 8.9 g of Thick & Easy powder in 236 mL of distilled water for 30 sec. The vehicle was left undisturbed for 5 min and stirred. The vehicle was mixed prior to use.

Twenty BDQ 100 mg tablets were ground into a fine powder using a glass mortar and pestle and mixed well with sodium saccharin 125 mg, citric acid 125 mg, potassium sorbate 100 mg and methylparaben 100 mg. Distilled water 20 mL was added and mixed to form a smooth paste. An additional 30 mL of distilled water was added and mixed, followed by 44 mL of Thick & Easy vehicle. The contents were mixed well to obtain 100 mL of 20 mg/mL of a uniform BDQ suspension. The suspension was transferred into an amber 120-mL pharmacy dispensing bottle with a syringe adapter and child-resistant cap. Step-wise preparation instructions are described in Table 2.

**Table 2:** Instructions for the preparation of BDQ 20 mg/ml formulation in a sugar-free vehicle

	Composition:	
Thick & Easy Sugar-Free Vehicle^*^		
Ingredient	Strength	Quantity
Thick & Easy	—	11.5 g
Distilled water	—	236 mL
BDQ sugar-free formulation,20 mg/mL, 100 mL^†^		
Ingredient	Strength	Quantity
BDQ tablets	100 mg	20
Methylparaben	100 mg	
Potassium sorbate	100 mg	
Citric acid	125 mg	
Sodium saccharin	125 mg	
Distilled water	50 mL	
Thick & Easy vehicle	44 mL (qs 100 mL)	

^*^ Instructions for preparing Thick & Easy^®^ sugar-free vehicle: 1 Measure 236 mL (8 oz) of distilled water into a suitable container 2 Add 11.5 g of Thick & Easy powder 3 Mix well with a spoon for 30 seconds and let the mixture sit for at least 5 min before use 4 Mix again just prior to use

^†^ Instructions for preparing 100 mL of 20 mg/mL BDQ sugar-free formulation: 1 Grind 20 tablets of BDQ (100 mg each) to a fine powder in a glass mortar and pestle. 2 Add 100 mg of methyl paraben, 100 mg of potassium sorbate, 125 mg of citric acid and 125 mg of sodium saccharin to the ground tablets in the mortar and mix all the powders well with the pestle 3 Add 20 mL of distilled water into the mortar using an oral syringe and mix the powder with a pestle to form a uniform suspension 4 Add an additional 30 mL water using an oral syringe and mix to form a uniform suspension. 5 Using an oral syringe, transfer 44 mL of Thick & Easy sugar-free vehicle to bring total volume in the mortar to 100 mL and mix to form a uniform suspension 6 Transfer the final contents from the mortar into an appropriately sized amber bottle

BDQ = bedaquiline.

### Storage stability of bedaquiline sugar and sugar-free formulations

Twelve bottles of each BDQ formulation were prepared as described above. Six bottles were stored at room temperature (22–24°C) and the remaining six bottles at 30°C for 30 days. For each storage condition, three bottles were designated for potency testing, one bottle for appearance and pH testing, one bottle for microbial testing; the extra bottle was to be used as backup, if needed. Aliquots were withdrawn from each bottle at the designated time points for performing the different tests.

### Outcome measures

The criteria for acceptable liquid formulations of BDQ included ease of preparation of the two formulations with the use of widely accessible and affordable ingredients in LMICs. Appropriate storage conditions were defined as the use of readily available plastic prescription bottles to be kept at ambient room temperature and at 30°C. The formulations were considered to be homogeneous and have acceptable potency if the measured BDQ concentrations were within 10% of the theoretical concentration during the study period.^12^ A smooth, uniform and off-white suspension with no lumps or clumps on visual inspection and no change in visual or microscopic appearance or in pH during the study period was considered an acceptable formulation. Conformance with the United States Pharmacopeia (USP)<1111> limits for aqueous oral liquids for total aerobic microbial counts (TAMC) and total yeast and mold counts (TYMC), and absence of specified microorganisms tested in the formulations during the study period were considered acceptable.

### Appearance and pH

From each bottle of BDQ suspension, 25 mL of the well-mixed suspension were transferred to a 30-mL beaker and examined by visual inspection to document any changes in appearance during the 30-day study period. A sample of each suspension was also placed on a glass microscope slide to observe and photograph any changes in appearance under a light microscope at both 40X and 100X magnifications. Appearance was documented on Days 0, 15 and 30.

The pH of the two BDQ formulations was measured using a digital pH meter calibrated with pH 4 and pH 7 buffers. The pH was determined on Days 0, 15 and 30.

### Measurement of bedaquiline potency

A stability-indicating high-performance liquid chromatographic (HPLC) method was developed and verified by conducting forced degradation studies on BDQ that included exposure to light, heat, acid, base and oxidation. The method was validated for reproducibility, system stability, linearity, specificity, ruggedness, filter suitability and working standard stability, and used to determine potency of BDQ in the two formulations on Days 0, 15 and 30. Three aliquots were withdrawn from each of the three bottles for a total of *n* = 9 at each time point.

The diluent was prepared with water and acetonitrile (1:1), and allowed to equilibrate at room temperature. The stock standard solution was prepared by dissolving 60 mg of BDQ fumarate bulk powder in the diluent in a 100-mL volumetric flask for a 0.5 mg/mL (as BDQ). The working standard solution was prepared by diluting the stock standard solution with diluent to a concentration of 0.02 mg/mL. The stock sample solution was prepared by volumetrically diluting 5 mL of BDQ (20 mg/mL) suspension to a concentration of 0.5 mg/mL in diluent. A 4-mL aliquot of this mixture was further diluted with the diluent to 100 mL for a concentration of 0.02 mg/mL. An aliquot of this mixture was filtered through a 0.45 μm polypropylene membrane syringe filter and the filtrate was analyzed by HPLC. Details of the HPLC method are provided in Table 3.

**Table 3:** Details of stability-indicating HPLC method for potency of bedaquiline formulations

Parameter	Details
Instrument	Hitachi L-2100 pump (Tokyo, Japan), Shimadzu (Kyoto, Japan) SPD-10AVP detector, Shimadzu SCL-10AVP controller, Shimadzu CTO-AVP column heater, SRI Instruments PeakSimple chromatography data system (Torrance, CA, USA)
Column	Waters Sunfire® (Dresden, Germany) (C18, 4.6 mm × 100 mm, 3.5 μm)
Wavelength	225 nm
Column temperature	40°C
Flow rate	1.5 mL/min
Injection volume	10 μl
Syringe filter	Polypropylene, 0.45 μm
Stock standard concentration	0.5 mg/mL
Working standard concentration	0.02 mg/mLW
Working preparation diluent	Water: acetonitrile (50:50)
Mobile phase	Water: acetonitrile: TFA (500:500:1)
Run time	6 min
Peak	4.5–5.5 min
Integration	Peak area method using PeakSimple data acquisition system, 5 Hz

HPLC = high-performance liquid chromatography; TFA = trifluoroacetic acid.

Ten μL of working standard solution was injected into the chromatographic system and the area under the BDQ peak eluting at 4.5 to 5.5 min was recorded. Ten μL of the diluent was injected as a blank to ensure no interference occurred with the BDQ peak. Five consecutive injections of the working standard were made to show the reproducibility of BDQ peak areas. Ten μL of the sample solution were injected into the chromatographic system, and the area under the BDQ peak was recorded as a percentage of the theoretical concentration. The working standard solution was injected periodically, throughout the run and at the end of the run to demonstrate HPLC system stability. The BDQ peak area response agreed with the system suitability average within 2%.

### Evaluation of microbial growth

Suitability studies were conducted to evaluate and verify the procedures described in the US Pharmacopeia USP<60>, USP<61> and USP<62> were suitable to recover the specific microorganisms in the two formulations. The verified procedures were used to enumerate and determine the absence of microorganisms in the two BDQ suspensions at Days 0, 15 and 30. The specific microorganisms tested were *Staphylococcus aureus* (American Type Culture Collection [ATCC] 6538), *Pseudomonas aeruginosa* (ATCC9027), *Candida albicans* (ATCC10231), *Escherichia coli* (ATCC8739), *Aspergillus brasiliensis* (ATCC16404), *Burkholderia cepacia* (ATCC25416), and *Zygosaccharomyces rouxii* (ATCC28253), which included those tested for extemporaneous vehicles.^13^ One mL of the sample from the BDQ suspension bottle was diluted with 9 mL of the neutralizing broth and used as positive control; 10 mL of the neutralizing broth alone was used as negative control. The inoculum of each pathogen was prepared using the phosphate buffer and serial dilution made with Tryptic Soy Broth (TSB; Alpha Biosciences, Baltimore, MD, USA). A volume of 0.1 mL of the selected concentration of 10^3^ colony-forming unit (cfu)/mL was spiked separately into the sample and positive control tubes and mixed well. Negative control tubes were spiked with 0.1 mL of TSB and mixed well. One mL of the spiked sample, positive control and negative control were placed on agar plates (bacteria on Tryptic Soy Agar [TSA] plates, and yeast and mold on Sabouraud Dextrose Agar [SDA; Alpha Biosciences] plates). The TSA plates were incubated at 30–35°C for 3–5 days and SDA plates at 20–25°C for 5–7 days. The colonies for each microorganism were then counted and the recovery percentage was calculated according to USP acceptance criteria (50–200%).

For specific microorganisms, appropriate plates were streaked and incubated per USP<62> following preenrichment at the specified temperatures for specified times. After incubation, the presence or absence of the target organism was verified by comparing against positive controls (Supplementary Data).

### Ethics statement

As this was not a human subjects study, ethics statement was not required for this study.

## RESULTS

Two uniform and smooth suspension formulations of 20 mg/mL BDQ were conveniently prepared in syrup and sugar-free vehicles and stored in standard amber plastic prescription dispensing bottles at room temperature and at 30°C. The suspensions could be easily re-dispersed with gentle shaking. The visual and microscopic appearance were off-white in color with no lumps of the tablet core present. The appearance of the two suspensions did not change during the entire 30-day study period at either temperature. As shown in Table 4, the potency of the sugar and sugar-free BDQ formulations ranged from respectively 99.1–101.1% and 97.7–99.9% of the theoretical concentration over the 30-day storage period, demonstrating that the aliquots withdrawn were homogeneous. The pH ranged from 4.46 to 5.44 in the sugar formulation and from 3.95 to 4.38 in the sugar-free formulation of BDQ.

**Table 4:** Potency of bedaquiline in two liquid formulations

Study day	Theoretical concentration (*n* = 9)			

	Sugar formulation		Sugar-free formulation	
	
	Room temperature Mean % ± SD	30°C Mean % ± SD	Room temperature Mean % ± SD	30°C Mean % ± SD
0	99.11 ± 1.03	99.14 ± 0.51	99.79 ± 0.84	99.56 ± 0.45
15	100.32 ± 0.93	100.28 ± 0.69	97.72 ± 0.88	98.97 ± 1.30
30	100.24 ± 0.53	101.14 ± 0.54	98.09 ± 1.45	99.87 ± 0.42

SD = standard deviation.

Microbial tests for six specific microorganisms and *Burkholderia cepacia* complex (BCC) showed no growth during the 30-day study period in either of the two formulations at room temperature and at 30°C. TAMC and TYMC met the USP<1111> limits for aqueous oral liquids during the entire 30-day study period in the sugar-free formulation and for 15 days in the syrup formulation at each storage temperature (Table 5). On Day 30, however, both the TAMC and TYMC exceeded the acceptable USP limits in the syrup formulation stored at room temperature and at 30°C. Therefore, this formulation was acceptable for storage up to Day 15 but not Day 30.

**Table 5:** Results of microbial limits tests of two bedaquiline liquid formulations

Microbial counts	Microbial limits test results^*^					

	Sugar formulation			Sugar-free formulation		
	
	Day 0 cfu/g	Day 15 cfu/g	Day 30 cfu/g^†^	Day 0 cfu/g	Day 15 cfu/g	Day 30 cfu/g
TAMC (room temperature)	<10	<10	2295	<10	<10	<10
TAMC (30°C)	<10	<10	955	<10	<10	<10
TYMC (room temperature)	<10	<10	955	<10	<10	<10
TYMC (30°C)	<10	<10	1110	<10	<10	<10

* Per USP<1111>, acceptable criteria for microbiological quality of oral aqueous liquids are TAMC <200 cfu/g and TYMC <20 cfu/g, and absence of *E. coli*.

^†^ Sugar formulation failed microbial quality criteria for TYMC on day 30 at room temperature and 30°C.

cfu = colony-forming unit; TAMC = total aerobic microbial counts; TYMC = total yeast and mould counts; USP = United States Pharmacopeia.

## DISCUSSION

BDQ has been approved in many countries as a 100 mg tablet and more recently by the FDA as a 20 mg dispersible tablet formulation, for administration daily for 2 weeks, followed by three times per week up to 24 weeks.^4^ As per the dosing guidelines, up to 10 dispersible tablets need to be dispersed for dose administration.

We followed the American Society of Health-System Pharmacists^14^ and USP^15^ guidelines for compounded aqueous non-sterile preparations. The selection of the preservatives was based on the recommended concentrations from literature^16^ and commercially available compounding vehicles.^17^ This has been a standard approach in the published stability studies of oral liquids to be used in pediatric patients in the peer-reviewed pharmacy journals. We conducted the stability studies for 30 days to meet the beyond-use date (BUD) requirements for USP<795>.^18^ The assigned BUD was confirmed by stability testing of potency, microbial count and absence of specific organisms.

We performed this study to develop a feasible BDQ dose administration alternative for children and older patients with dysphagia, primarily for settings in which dispersible tablets are not available. These formulations are ready to administer and avoid the need for dispersion preparation at the time of administration of each dose for 24 weeks.

BDQ 100 mg tablets in immediate-release dosage form are designed to disintegrate and release the drug quickly. There are no rate-controlling features in the formulation. Therefore, these tablets lend themselves well to a compounded formulation. Svennson et al. have demonstrated that there was no significant difference in the bioavailability of crushed suspended tablets and the whole tablets.^19^

Two extemporaneous oral liquid formulations of BDQ (sugar and sugar-free) were developed. The concentration of 20 mg/mL would permit dosing small children with a limited but accurately measurable volume. The sugar-free liquid formulation would serve the needs of patients with sugar intake restrictions. These formulations can be used effectively for dose titration, especially in young children, and in patients with dysphagia. No dose preparation is required prior to administration. We investigated the stability of the formulations at two different temperatures to accommodate the different storage temperatures expected in the field. Simple syrup is 85% w/v or 65% w/w sucrose solution per the monographs for simple syrup in all pharmacopoeia. As undiluted simple syrup has self-preserving properties, we did not add preservatives to this formulation. Cane sugar is readily sourced in LMICs at a low cost. For the sugar-free formulation, we used a commonly available instant food and beverage thickener, Thick & Easy, a modified food starch, which is easy to work with and can be sourced in high TB burden countries. The selection of Thick & Easy was confirmed with pharmacists and social scientists working with these patients. As the sugar-free preparation contains food starch and water and the goal was to use the suspension for at least 2 weeks, it was necessary to add preservatives. The concentration and safety of preservatives used here have been well established for use in oral formulations.^14^

The two liquid preparations of BDQ for oral administration met the requirements of desirable compounded formulations. Stability-indicating methods were designed to test these suspensions and identify any degradants. Our results showed that BDQ (20 mg/mL) was stable in simple syrup and sugar-free liquid formulations after preparation and storage at both room temperature and 30°C. The microbial count did not change for the sugar-free formulation during the 30-day storage period. However, the simple syrup formulation did show growth at Day 30, although it met the outcome measures for microbial growth at 15 days. The use of potassium sorbate and methylparaben as preservatives in the sugar-free liquid formulation ensured no substantial growth of microorganisms during the storage period of 30 days. Citric acid was added to the sugar-free formulation to maintain the pH below 6, which is optimum for antimicrobial activity of potassium sorbate.^16^ Growth of microorganisms in the simple syrup formulation on Day 30 can be explained by the lack of preservatives in the formulation. The sugar-free liquid formulation provides a choice for patients with diabetes or on calories restriction.

We purposefully utilized easily accessible and affordable ingredients (e.g., cane sugar, Thick & Easy) to prepare the formulations to maximize their use in LMICs. Another alternative is to use commercial simple syrup when available and affordable. The BDQ liquid formulations were pharmaceutically elegant, and no noticeable changes occurred in appearance or the potency of these formulations. Flavors and sweeteners can be added to enhance the organoleptic properties after conducting appropriate stability studies.

The plastic bottles used for storing the suspensions were representative of the prescription dispensing bottles routinely used in pharmacies, including in LMICs. These formulations have to be prepared in a compounding pharmacy. It is our expectation that the patient would be able to get the compounded preparation during routine follow-up visits to the clinic.

## CONCLUSIONS

BDQ liquid formulations in simple syrup and sugar-free suspensions were easily prepared with widely available and affordable ingredients. These formulations will support the practical effective dosing in children and in patients with dysphagia. BDQ was chemically and physically stable during the 30-day study period at room temperature and 30°C. Microbial counts met the USP limits for 30 days in the sugar-free formulation, but for only 15 days in the syrup formulation. Thus, liquid formulations of BDQ can be extemporaneously prepared and stored for 15 days in simple syrup and 30 days in the sugar-free formulation.
